# Ruminations on sustainable and safe food: Championing for open symbiotic cultures ensuring resource efficiency, eco‐sustainability and affordability

**DOI:** 10.1111/1751-7915.14436

**Published:** 2024-03-11

**Authors:** Ugo Javourez, Silvio Matassa, Siegfried E. Vlaeminck, Willy Verstraete

**Affiliations:** ^1^ TBI, Université de Toulouse, CNRS, INRAE, INSA Toulouse France; ^2^ Department of Civil, Architectural and Environmental Engineering University of Naples Federico II Naples Italy; ^3^ Department of Bioscience Engineering, Faculty of Science University of Antwerp Antwerpen Belgium; ^4^ Center for Microbial Ecology and Technology (CMET), Faculty of Bioscience Engineering Ghent University Gent Belgium

## Abstract

Microbes are powerful upgraders, able to convert simple substrates to nutritional metabolites at rates and yields surpassing those of higher organisms by a factor of 2 to 10. A summary table highlights the superior efficiencies of a whole array of microbes compared to conventionally farmed animals and insects, converting nitrogen and organics to food and feed. Aiming at the most resource‐efficient class of microbial proteins, deploying the power of open microbial communities, coined here as ‘symbiotic microbiomes’ is promising. For instance, a production train of interest is to develop rumen‐inspired technologies to upgrade fibre‐rich substrates, increasingly available as residues from emerging bioeconomy initiatives. Such advancements offer promising perspectives, as currently only 5%–25% of the available cellulose is recovered by ruminant livestock systems. While safely producing food and feed with open cultures has a long‐standing tradition, novel symbiotic fermentation routes are currently facing much higher market entrance barriers compared to axenic fermentation. Our global society is at a pivotal juncture, requiring a shift towards food production systems that not only embrace the environmental and economic sustainability but also uphold ethical standards. In this context, we propose to re‐examine the place of spontaneous or natural microbial consortia for safe future food and feed biotech developments, and advocate for intelligent regulatory practices. We stress that reconsidering symbiotic microbiomes is key to achieve sustainable development goals and defend the need for microbial biotechnology literacy education.

## Graphical abstract

we propose to re‐examine the place of spontaneous or natural microbial consortia for safe future food and feed biotech developments. Microbiomes can already adequately be managed, and provide high‐quality food and feed commodities through emerging microbial biotech, but symbiotic fermentation is currently overlooked compared to axenic production. We stress that reconsidering symbiotic microbiomes is key to achieving sustainable development goals, and defend the need for microbial biotechnology literacy education as well as intelligent regulatory practices.

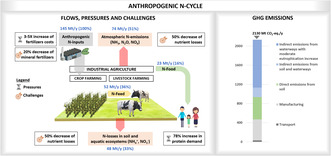

## MICROBIAL BIOTECH AND PROTEINS: QUID HOC

Microorganisms like bacteria, yeasts, filamentous fungi, eukaryotic microalgae and protozoa are powerful upgraders of rather simple bulk substrates to higher value products. Microbes, and particularly aerobes, can grow at doubling times of a few hours, converting feedstock to protein and other valuable metabolites at rates in the range of 10–200 g dry matter per litre volume and per day (Huang & Reardon, 2022; Matassa et al., 2022). They thereby can achieve yields which surpass those of higher organisms, including insects and livestock, with a factor 2 to 10 (see Table 1). A large part of their performance is attributable to their cooperation and communication as part of consortia or microbiomes, illustrating the principles of the so‐called market economy (Tasoff et al., 2015). Trading positively with one another, multiple microbial strains maximize the use of available resources under the given environmental (thermodynamic) conditions compared to single species. Digestive tracts provide the first set of examples for the efficiency of microbial consortia, (co‐)evolving over millions of years to make the most of the specific ingested menu. Animals which are only fed on cellulose‐rich, slowly degradable types of biomass present digestive tracts with microbial compartments much larger (e.g. bovine rumen) than animals fed on easily degradable nutrients such as starch or proteins (Godon et al., 2013). Such digestive tracts achieve organic matter degradation rates surpassing state‐of‐the‐art anaerobic digestion facilities by a factor from 10 to 20 times upwards (Weimer, 2022). Remarkably, the basic explanations for this have yet not been unequivocally established and could be related to the ecology and metabolism of protozoa, highly present in the rumen but not in artificial reactor systems.

**TABLE 1:** Conversion efficiencies of higher and lower organisms into food.

Bioconversion system	Specific type of organism (group) and/or product	PCE or NCE: Protein or nitrogen conversion efficiency (kgN_out_ per kgN_in_)	FEE: Feed energy to edible energy conversion efficiency or feed‐COD to biomass‐COD conversion efficiency (kcal_out_ per kcal_in_ or kg COD_out_ per kg COD_in_)
Terrestrial animals	Cow – Beef	1%–9%	1%–7%
	Cow – Milk	9%–25%	5%–27%
	Chicken – Broiler meat	20%–54%	9%–25%
	Chicken – Eggs	4%–39%	6%–19%
	Pig – Swine meat	7%–41%	8%–32%
Aquatic animals	Salmon	18%–28%	12%–25%
	Shrimp	5%–25%	7%–9%
Insects	*Black soldier fly* larvae	8%–80%	22%*
	Yellow mealworm	22%–58%	33%*
Bacteria (excl. cyanobacteria)	Aerobic heterotrophic bacteria (high rate)	80%–100%	55%–75%
	Hydrogen‐oxidizing bacteria (aerobic)	80%–100%	30%–32%
	Methane‐oxidizing bacteria (aerobic)	80%–100%	23%–27%
	Purple non‐sulphur bacteria (both photo‐heterotrophic and photo‐hydrogenotrophic strains)	80%–100%	100%**
	‘Spirulina’: *Arthrospira* sp. or *Limnospira* sp. (photoautotrophic cyanobacterial microalgae)	76%*	N/A
Eukaryotic microalgae	*Chlorella* sp. (mixotrophic)	75%*	36%–67%
Fungi	Aerobic *Saccharomyces cerevisiae* (baker's yeast)	80%–100%	30%–40%
	Anaerobic *Saccharomyces cerevisiae* (baker's yeast)	80%–100%	8%–10%
	*Fusarium* sp. (filamentous; mycoprotein)	25%–65%	25%–30%
	*Pleurotus* sp. (mushroom)	33%–39%	23%*

*Note*: Unless stated, the values have been benchmarked in Javourez et al. (2021) which detailed the data selection and references mobilized. For microorganisms, the notion of ‘feed’ does not make much sense, so only nitrogen and energy conversion efficiencies are presented, and not the commonly used ‘feed conversion ratio’, which can reach dispersed values depending on normative choices on feed and product moisture content, among others. For high‐rate aerobic heterotrophic bacteria (Papini et al., 2023), purple bacteria (Alloul et al., 2019; Spanoghe et al., 2021) and baker's yeast, the nitrogen conversion efficiency is assumed the same as for hydrogen and methane‐oxidizing bacteria, as these organisms all have low nitrogen affinity constants, and the energy conversion efficiency is estimated based on the chemical oxygen demand (COD) yield. *: based on only one study; **: in theory, when all input COD or H_2_ is converted into biomass with the support of the phototropic route; N/A: not applicable as a pure photoautotrophic route.

Microbial consortia are historically and traditionally, up till today, commonly used to produce edible ingredients for food and feed applications in the form of so‐called ‘open’, ‘spontaneous’, ‘terroir‐based’, ‘natural’ or even ‘symbiotic’ fermentation. The latter illustrates the fact that organisms work coherently together to achieve the overall effective conversion. This is the case with beer, wine, cheese, kefir, kombucha, sauerkraut, sourdough, cacao, olives, silage, etc. Foods based on natural fermentation processes have been produced for centuries and are present in almost all gastronomies of the world, as recently visualized by Gänzle (2022) in his ‘periodic table of fermented foods’. Provided care and crafts(wo)manship, such microbial biotech processes are reliable and stable, and supply safe commodities with improved nutritional, organoleptic and shelf‐life properties. The European Union (EU) is probably the most challenging region in the world to enter with new food products on the market, linked to the Novel Food regulations implemented by the European Food Safety Agency (EFSA). While food safety is paramount, and the precautionary principle is commendable, in practice, this approach is felt to be a hurdle for novel types of fermentations, especially when they are based on spontaneous natural processes, most probably based on the lack of experience and guidance in relation to new symbiotic ferments. This current ambiguity constitutes a strong hindrance towards the development of a variety of new symbiotic fermentation processes in the EU and should be addressed as soon as possible.

In this context, a new generation of microbial biotech is following an alternative route, building on the axenic bioreactors typically based on one single natural or engineered strain. Using pure cultures to produce ingredients for the food chain, is often termed ‘biomass fermentation’ when the whole cells of a natural strain are used as proteinaceous ingredient (single‐cell protein), and ‘precision fermentation’ when a genetically modified organism produces and excretes a target protein or other compound (Specht & Crosser, 2020). In terms of cost per kg product, the approach with aseptic bioreactors compared to open symbiotic fermentation increases substantially capital and operational expenditures, as pure cultures impose high sterility requirements, more specialized feedstocks, more specific environmental controls, more complex scale‐ups, etc. Furthermore, they have a lower substrate‐to‐product bioconversion efficiency, in other words, more input is needed per output, and more waste should be managed (Javourez et al., 2023). Additionally, for precision fermentation, downstream processing demands are higher (e.g. protein purification). These combined aspects would logically yield a larger (hence worse) environmental footprint when producing with defined cultures compared to using open symbiotic cultures. When axenic biomass or precision fermentation is targeted for feed or food applications in practice, one should ideally focus on products that go beyond the basic nutritional aspects. Properties that add value can be functional, relating to health benefits (nutraceuticals), technical physical/chemical features and sensory properties, among others. Clearly, economies of scale are required to be commercially viable. Existing or planned production facilities target 20–40,000 tons of protein‐rich commodities per year based on 100–250 m^3^ bioreactors, of the same magnitude as the current established fungi‐producing Quorn facility (Banks et al., 2022). Overall, the diversity and frequency of food‐related axenic microbial biotech patents are ever‐increasing (Nyyssölä et al., 2022), with technologies relying on gas fermentation largely put forward (Woern & Grossmann, 2023; Xu et al., 2023). On the other hand, symbiotic open fermentation, albeit with a longer culinary tradition, demonstrated feasibility at any scale and with at least as much innovation potential, is still largely underrepresented in microbial biotech investments and development directions (Specht & Crosser, 2020). When targeting food and feed commodities, most studies related to microbial biotech focus on single strains or, at most, on the simultaneous or sequential culture of two strains (Javourez et al., 2021).

## BROKEN FOOD SYSTEMS

Going back to microbial biotech as a whole, it is important to unravel the main rationales and stakes behind the developments, capture future directions and understand what agri‐food system stakeholders care about, from feed producers and livestock owners, over veterinarians and nutritionists, to food companies and food risk assessors and regulators. Indeed, microbial biotech has the potential to either escort or challenge the place of animal production in the ongoing transition towards sustainable food systems.

In fact, mankind has struggled over the centuries with the development of new foods and feeds, as described in ‘Meals to Come: a History of the Future of Food’ (Belasco, 2006). The author distinguishes a philosophical duality going throughout history: on the one hand, Malthus‐inspired thinkers with a dystopian mindset tend to distrust new developments. On the other hand, Condorcet‐related thinkers with a utopian perspective advocate that technology will provide for all the necessary food and feed in abundant (cornucopian) amounts. Microbial food and feed biotech are part and parcel of this debate, as most of its historical development rationale (initiated in the 60s) builds on the need to find alternative protein sources, as cyclically put in the global spotlight due to Malthusian analysis or the development of regenerative life support systems for long‐term space missions (Clauwaert et al., 2017).

**FIGURE 1:**
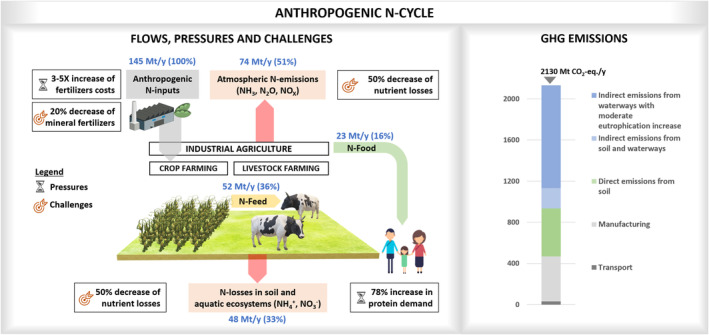
Main flows, pressures and challenges (EU Green Deal targets) linked to the anthropogenic nitrogen cycle. Adapted from Matassa et al. (2023). The left panel shows some of the flows linked to the anthropogenic nitrogen cycle, along with the main pressures and challenges arising from international policies (EU Green Deal) and socio‐economic dynamics (fertilizers production cost, protein demand). N‐inputs include: Haber–Bosch process (100 Mt/y), biological fixation in crops (35 Mt/y) and deposition in animal rearing (10 Mt/y). N‐emissions include: volatilization from the field (48 Mt/y), loss/volatilization from manure storage (26 Mt/y). N‐food includes: vegetable (13 Mt/y) and animal (10 Mt/y) protein sources. The right panel shows the global estimated GHG emissions (Mt CO_2_‐eq./y) linked to current artificial nitrogen fertilizers production and utilization, including: transport (29.8), manufacturing (438.5), direct emissions from soil (465.9), indirect emissions from soil and waterways (196.4) (Drugmand et al., 2022). The indirect emissions from waterways with moderate eutrophication increase (1000 Mt CO_2_‐eq./y) refer to the potential future increase in current GHG emissions from lakes and impoundments due to global eutrophication phenomena (DelSontro et al., 2018).

More recently, food systems and particularly the supplies of animal‐based proteins have increasingly been pointed at for their large contribution to the global environmental burden (ca. 30%–35% of greenhouse gas emissions; GHG) as well as their key responsibility to overshooting safe and just Earth system boundaries (Gerten et al., 2020). In the first place, global livestock production systems, providing around 40% of the protein (among other nutrients), are estimated to generate more than half of total food system's GHG emissions and to rely on three‐quarters of total arable lands (IPCC, 2019; Xu et al., 2021). Moreover, current food systems are heavily dependent on fossil fuels, not only due to mechanization and global supply chains, but also due to nitrogen fertilizer input requirements. Nitrogen (N) is an essential element in all proteinaceous commodities but is still largely supplied by the Haber‐Bosch process (producing more than a third of total reactive nitrogen; Spiller et al., 2022). Producing 1 kg of reactive N from dinitrogen present in the air is equivalent to around 2–3.5 kg CO_2_, and globally represents 1%–1.4% of the global GHG emissions (Matassa et al., 2023; Mayer et al., 2023). Even though progress on eco‐friendlier NH_3_ production with blue or green H_2_ has been industrially announced, it may take several decades before these routes become economically viable (Mayer et al., 2023). Upon application of fertilizers, efficiency gaps are present all along the nutrients‐to‐farm‐to‐fork value chains. The nitrogen use efficiency (NUE) of a food system can be defined as the ratio of nitrogen consumed by citizens through food and fibres to the quantity of virgin or synthetic nitrogen introduced into the system. For example, the current European NUE was recently estimated to be lower than 20% (Leip et al., 2022), and is for a livestock‐intensive region like Flanders, for instance, as low as 11% (Vingerhoets et al., 2023). Such gaps are mostly due to limited use of input N by crop systems (the rest is lost by run‐off, leaching, etc.), limited conversion by animal production systems (see Table 1), many food losses and wastes along the chain (Figure 1) and underexploited potential of nutrient recycling. Such low NUE leads to undesired negative impacts on the environment and human health. Diffuse and point source emissions to air, soil and water bodies lead to biodiversity loss, emissions of N_2_O as powerful GHG and health concerns (e.g. particulate matter). Even dedicated treatment of reactive nitrogen, for instance through nitrification/denitrification in wastewater, is associated with an environmental footprint of around 5 kg CO_2_ equivalents per kg N, linked to the energy input and partial emission as N_2_O (Spiller et al., 2022). In the end, in our biosphere, the nitrogen cycle is responsible for ca. 6% of all global warming (Figure 1). Many technical solutions have been proposed to limit N leaks and increase N recycling. Examples include limiting volatilization from livestock manure through acidification and recovering nitrogen from wastewater streams through stripping/scrubbing. However, it is still to be demonstrated whether their wide implementation truly offers long‐term solutions to food systems with constant reactive nitrogen demand without downsizing virgin nitrogen production. This uncertainty also arises because many of these technologies present lower efficiencies and higher energy requirements than the Haber‐Bosch process (Leip et al., 2022; Spiller et al., 2022).

Food system concerns are not limited to the environmental dimension: the notion that conventional livestock production is ethically questionable is constantly on the rise, as attested by the numerous political parties and civil associations focusing on this issue. Yet, the environmental and ethical dimensions are also hardly reconcilable at the global scale at constant livestock production rates, as, for example, more extensive production usually leads to lower feed conversion efficiencies (Council for Agricultural Science and Technology, 1999).

Accordingly, the place of animal‐based food in future diets and their adequation with environmentally and ethically concerned society are largely debated. There is a general agreement on the urgency of global downsizing of livestock and, more particularly, ruminant‐based production systems. This is seen as necessary to mitigate environmental impact while supplying enough food to a growing population. However, the details on how, where and to what extent such livestock production should be downsized remain a subject of passionate debate, fed with diverging views. This is further illustrated by the absence of clear and consensual roadmaps on what a sustainable food system should look like. In contrast, such roadmaps are established for other sectors, like energy systems. Indeed, existing prospective food system scenarios or assessments place different weights on demand‐side interventions, such as diet shifts and food waste reduction, and supply‐side solutions, including technology development and efficiency improvement. This leads to different trajectories towards sustainable food systems (Duru & Therond, 2023).

## THE PLACE OF MICROBIAL BIOTECH IN FUTURE FOOD SYSTEMS NARRATIVES

Interestingly, despite the plethora of microbial biotech patents and developing start‐ups, microbial biotech is still quite absent from the major authoritative discourses depicting future food systems (e.g. from FAO, EU Green Deal, Agrimonde‐Terra, etc.). For example, the reference foresight scenarios for Europe in 2040 published by the Joint Research Center (JRC) does mention the production of ‘synthetic foods’, yet only refers to cellular agriculture (that is, animal cells culture in axenic conditions to produce e.g. cultivated meat; Crosser et al., 2019) and considering this as a backup solution in case conventional agricultural production is not viable due to, for instance, harsh environmental conditions (Vesnic Alujevic et al., 2023). However, many microbial biotech projects position themselves both as mitigation and adaptation solutions to the many food system challenges. These could integrate strategies to reinforce food security and reduce disruption supplies risks (Denkenberger et al., 2017; Tzachor et al., 2021), but also could directly join the basket of environmental impact mitigation strategies at different levels of the food systems. These include the production of microbial fertilizers, replacing synthetic fertilizers (Spanoghe et al., 2020), of microbial proteins as feedstuffs, substituting conventional proteinaceous ingredients such as fishmeal or soybean meal, and of microbial proteins as food ingredients, mimicking the organoleptic properties and other functionalities of meat‐, dairy‐ or egg‐based foods (Pikaar et al., 2023). All these approaches propose to simultaneously tackle the main challenges previously described: reducing environmental impacts including a solution for the nitrogen dilemma (Matassa et al., 2023), dealing with ethically questionable animal production and positively contributing to human health. Presented as highly disruptive win‐win solutions with limited trade‐offs, the idea of microbial meals slowly infuses outside the sci‐fi narratives into the mainstream public (Tubb & Seba, 2019).

## UNMATCHED RESOURCE USE EFFICIENCIES OF MICROBIAL BIOTECH

Resource use efficiency strongly influences the environmental and economic sustainability of protein production processes. The main rationale supporting microbial biotech over the conventional animal‐based route is its efficiency in upgrading substrate energy and nutrients to proteins. In conventional bioconversion systems (such as livestock production), only a small share of input energy and nitrogen is converted to an edible output; the rest is wasted and dispersed in the environment. At best, livestock production converts 50% of input nitrogen into edible proteins, and 30% of input energy into edible calories, which are similar to the values achieved by insects farming (Table 1). Yet, microbe‐driven ruminant systems uniquely convert humanly inedible or non‐nutritional compounds into edible commodities: nitrogen, such as urea and other non‐protein nitrogen sources, and calories, in the form of cellulose and hemicellulose. However, this process is characterized by a low conversion efficiency, leading to subsequent large environmental impact, linked to increased requirements for land area, freshwater, fertilizers applications, etc., topped up with co‐production of CH_4_ as potent GHG. Consequently, ruminant meat is probably the most polluting constituent in our global diet (Searchinger et al., 2018).

When applied in a bioreactor, on the other hand, microorganisms can achieve extremely high resource use efficiencies. Not only can microbes have low‐affinity constants for carbon, nitrogen, phosphorus, etc., but they also convert these nutrients into biomass with high efficiency. For instance, microbes can theoretically convert reactive nitrogen into edible proteins at a near 100% efficiency, while more than 80% efficiency is already achieved in practice (Pikaar, Matassa, Rabaey, et al., 2018). Moreover, when calling on biological nitrogen fixation, i.e. the microbially‐mediated conversion of inert nitrogen gas into reactive nitrogen, microorganisms could ideally be independent of synthetic nitrogen supply and NUE values can reach in principle values (far) above 100%, yet at the expense of productivity. For carbon, we define the carbon use efficiency (CUE) as the ratio of output carbon in biomass to input carbon in the bioreactor, a parameter strongly depending on the chosen combination of feedstock(s), carbon type, energy source and suitable microorganism(s) (Table 1). As defined, CUE cannot be higher than 100%. When organic carbon is used (in heterotrophic processes), the CUE can be calculated based on the chemical oxygen demand (COD), a standard parameter used in wastewater treatment, which can be seen as a proxy for the energetic content or calorific value. When inorganic carbon such as CO_2_ is used (in autotrophic processes), the CUE of both phototrophic and chemotrophic lifestyles can in principle approach 100%. Yet, lower CUE values are achieved by chemoorganoheterotrophs, as in this lifestyle carbon serves the role of energy source as well, next to its use as a carbon source. For the latter case, reported CUEs remain nonetheless superior to higher organisms, which also deploy this metabolic lifestyle (Table 1). Aerobic conversions give the highest biomass yields, with rapidly growing bacteria as CUE champions. For chemoorganoheterotrophs, biomass yield, CUE or FEE (for feed energy to edible energy conversion efficiencies) are all correlated, and reach their theoretical upper limit between 30% and around 60%, depending on the substrate (El Abbadi et al.,^2019^; Erickson et al., 2000; Mishra et al., 2020). On complex substrates like wastewater, the observed yield of high‐rate symbiotic cultures can even be higher due to biosorption, up to, for instance, maximum values up to 75% (Papini et al., 2023). Aerobically grown yeasts and filamentous fungi generally present lower CUE values (Table 1).

When estimating the overall resource use efficiency of a microbial protein bioreactor, also energy from additional sources should be considered. While phototrophic systems have very appealing CUE values, the energy needs are heavily impacted by the light source (sunlight vs. artificial light). Further, aerobic approaches need to be oxygenated, typically with compressed air and in hydrogenotrophic systems, energy is contained in the fed H_2_. Also, gas‐to‐liquid transfer efficiencies co‐determine the overall resource efficiencies of bioreactor solutions, linked to the dosing of O_2_, CO_2_, CH_4_, H_2_, NH_3_, N_2_, etc., as carbon source, electron donor/acceptor and nitrogen source, which should be maximized through tailored design and operation. Finally, besides conversion efficiencies, volume‐based productivities are of high importance, as, clearly, faster processes lead to smaller bioreactors and therefore lower capital expenditures.

To illustrate some of these efficiencies and rates: 1 kg of C_6_ sugars and ca. 40 g of reactive nitrogen can yield up to 400 g of dry yeast or fungi, which corresponds to around 150 g of crude proteins, achieved in 1 day and less than 1‐litre reactor volume in industrial conditions (Javourez et al., 2023). Even after processing losses and refining, such fungal culture converts 1 kg sugar input into around 1 kg of ready‐to‐eat meat‐analogue products (as Quorn's formulations). Bacterial cultures can achieve even higher growth‐rate performance in terms of volumetric productivity and produce up to 1 kg of dried bacterial meal per kilogram of methane or up to 3 kg per kilogram of hydrogen (Matassa et al., 2020). Covering the entire ‘waste‐to‐nutrition’ value chain, microbial protein production could yield in the range of 20–200 kg proteins per ton of dry wood, straw or manure (to mention a few), depending on the specific conversion routes (Javourez et al., 2023). Such processes have the potential to unlock waste and lignocellulosic bioresources to enter the food chain and open the perspectives of massive land‐free protein supplies, given the availability of corresponding waste and residues (Chen et al., 2022). For example, France's residual biomass resource is estimated to host as much proteins as its current vegetable protein production and host as much sugars (majorly cellulosic in terms of calories) as a fourth of France's final energy consumption (Javourez et al., 2023). Yet most of it is currently left to decay on land, as 60% of the weight of this resource consists of straw and manure: this is an untapped potential to supply food systems.

## MICROBIAL BIOTECH TO UPGRADE RESIDUAL RESOURCES: EXAMPLES AND CHALLENGES

Using the power of microorganisms to directly loop back residual nutrients into the food chain has a long research tradition. Of course, strategies depend on the specific to‐be‐upgraded resource, both in terms of composition and in terms of safety risks (Javourez et al., 2021). Yet, despite its potential and scientific achievements, general public acceptance is not ready for these processes and a positive attitude by the industry and the regulator towards this potential is missing.

As a first example, the strong intensification of animal production in Flanders (Belgium) and subsequent excess pig slurry led to the development of recovery technologies already in the 1970s. A simple process consisted of aerating the liquor (slurry is ca. 10%_DM_ content) to directly develop microbial biomass, and excess nitrogen was further nitrified. Stabilization was obtained through acidification, obtaining a protein‐rich liquor treated with nitrites, somehow similar to salami production. Despite successful zootechnical trials (over several generations of sheep; Vanstaen et al., 1976) as well as scientific acclamations, the project was abandoned due to mismanaged communication with the public (tabloids bashing campaigns).

Implementing ‘clearing’ barriers, even if the risks are just perceived (and not technical), can help the acceptance. Indeed, manure and sludge are already commonly reused as fertilizing agents, meaning that the soil is considered an adequate risk clearance barrier to recycle faecal matter into food and feed (Verstraete et al., 2016). This is also the case for mushroom production, usually directly grown on a straw‐manure mixture. This perception of natural bodies as risk clearer is directly traduced into the legislation in the EU, driving the rationales of the EU Fertilizing Product Regulation and wastewater reuse frameworks. While reusing wastewater for drinking water production in France is prohibited, discharging the same wastewater into natural water bodies and taking it back a few meters downhill to produce drinkable water is allowed, even though most water (in terms of flow) consists of the same treated wastewater.

Building on this risk clearance concept, circular bioeconomy waste‐to‐nutrition microbial biotech projects then need to implement a sequence of unit operations of enough process intensity to ensure food safety and the perception of distance between the waste and the final edible ingredient (Javourez et al., 2023). This is, for example, the case of the conversion of residual C, N and P into single‐cell proteins through a sequence of bioconversion steps based on a purple bacterium and a cyanobacterium, developed in the late 1980s, now key components in the micro‐ecological life support system alternative (MELiSSA) developed by the European Space Agency (Walker & Granjou 2017). The continuous development of this faeces‐to‐food technology to support long‐term space missions illustrates that clarifying the constraints (here, rather extremes) and rationales behind such recovery projects can trigger acceptance and open the room for further diffusion of this technology from niche markets to other applications. This is not just an incantation, as some of these faecal upcycling projects are already widely established, such as bioflocs technology. By adding organic carbon to aquaculture ponds, microbes can produce cells upgrading the nutrients in the water which, when growing in flocs, can be grazed upon by aquatic animals and cycle back wasted nutrients to the upper trophic levels. Where implemented, this technology doubled aquaculture's NUE without adverse effects on the consumer acceptance (Matassa et al., 2023). Moreover, this technology has the advantage of in‐situ recycling without implementing the resource‐intensive sequence of drying and refining operations (see next section). Notably, feacally contaminated food items, such as the Kopi Luwak coffee beans, have already reached EU markets.

Closer to symbiotic fermentation principles, examples of microbial biotech developed to directly upgrade the nutritional content of low‐value streams are based on solid‐state fermentation (Javourez et al., 2021). These do not differ, in essence, from crop ensiling but target specific enrichments (e.g. in lipids, proteins) and are applicable to a wide span of co‐substrates provided these initially comply with feed safety standards (for example, a mixture of vegetable waste and straws, Dou et al., 2022). Yet, for streams with latent risks (e.g. manure, sludge), direct fermentation (either solid or liquid) for nutritional applications is not foreseen. Instead, biomass cracking to soluble organics or even to the gas phase (to unlock subsequent safe fermentation) seems required to ensure risk deconstruction, but at the cost of reduced conversion efficiency and increased pretreatment utility (Javourez et al., 2023).

## MICROBIAL BIOTECH DIFFICULTIES AND CRITIQUES

The weaknesses of microbial upgrading are evident and primarily relate to the current consumers' mindset. Moreover, as for the rest of the technical supply‐side solutions to fix food systems, concerns about the actual implementation plausibility of microbial biotech (e.g. cost competitiveness, etc.) as well as critics about their actual environmental and social impacts mitigation potential relevance have not been solved.

First of all, while single‐cell products (SCP) can have appealing essential amino acid profiles, they do not on all levels match the nutritional value nor other desired organoleptic or technofunctional properties of conventional food and feed ingredients. SCP constitutes a cocktail of proteins, next to some potentially interesting non‐protein compounds.

Competing with the mass production of soybean meal and other established, often subsidized, bulk protein production routes in the livestock sector is typically economically challenging for SCP. Despite the lack of major technical difficulties to scale common microbial biotech, these are still not reaching price equity with conventional feed ingredients, which delays their wide deployment. These microbial ingredients are currently estimated to reach the order of 1500–2500 euros per ton, thus in the range of quality fishmeal prices (El Abbadi et al., 2021). This is mostly because microbial cells are difficult to harvest (bacteria of ca. 1 μm; yeast‐fungi‐algae size around 5–10 μm), to dry and refine, which induce large capital expenditures (centrifuge, filtration, etc.) and power consumption to operate such downstream treatments (from 3 g per litre broth concentration or 0.3%_DM_ to 90%_DM_ stable powder). This consumption was estimated within the range of 2–5 kWh/kg_DM_ for microbial protein production. Yet, when including upstream pretreatments (in the case of residues sourcing or e.g. electrolysis to generate hydrogen), then the full microbial food and feed production pathway is likely to reach the consumption of 10–30 kWh/kg_DM_, apart from the other involved chemical and heat inputs (Javourez et al., 2023; Sillman et al., 2019). These utilities must be drastically cut down to unlock plausible large‐scale deployment of microbial biotech. Indeed, under current forecasted utilities, phasing out French soybean meal imports through microbial protein production was estimated to increase the power intensity of food systems in France by a factor 2 to 10 (Javourez et al., 2023).

Therefore, for SCP to enter food and feed markets more successfully, research and development should focus on creating higher added value. This can be achieved by enhanced nutritional, health, technofunctional and organoleptic features. These enhancements could include vitamins, interesting fatty acids, antioxidants and constituents with prebiotic or immunostimulatory effects, among others. In animal feeds for products with colour (such as salmon, shrimps, egg yolk, etc.), pigments can increase the SCP value. Pet food may be an interesting higher value market to target. For food, ideally, specific types of SCP (or extracts thereof) have foaming, emulsification or gelling properties. Also, innovations in downstream processing and feed/food formulation towards palatability are welcomed, yielding attractive taste, odour, texture and appearance. For the moment being and particularly in the case of bacteria, the animal feed ingredient markets are mostly targeted. Efforts also focus on the cultivation of yeasts, filamentous fungi and algae, which display a more complex structure, enabling potentially higher integration rates in livestock feed formulations. But they should preferably be directly targeting human food markets.

One of the largest difficulties faced by microbial biotech is the stringent regulatory space in which it deploys. For instance, in Europe, EFSA recognizes traditional fermented products like mushrooms, sauerkraut and raw milk cheeses as safe and healthy parts of conventional diets. However, it categorizes all new symbiotic or axenic ferments as ‘novel food,’ as discussed in the first section. Therefore, to enter the European consumer market, these new developments must complete a dossier. This process incurs costs of millions of euros and necessitates years of testing to comprehensively demonstrate the absence of risks. In the requirements, the fact is mentioned that microbiome‐based foods need to be permanently guaranteed in terms of constancy of the microbiome composition, which is impossible as formulated in practice, even considering the recent advances in terms of fermentation monitoring and quality control of microbiomes (De Vrieze et al., 2020). Indeed, it is required that the member species of the microbiome are fully known at all instants, down to the last strain and that the strain distribution remains constant over time. Therefore, novel food innovations based on symbiotic microbial consortia are currently virtually blocked. Only axenic fermentation, that is, the culture of conventional (well‐established) specific microorganism strains (present in the Qualified Presumption of Safety List) such as the yeast *Saccharomyces*, the fungus *Aspergillus* and the alga *Chlorella* (EFSA Panel on Biological Hazards (BIOHAZ) et al., 2023), grown in ‘pure’ culture under strict axenic conditions, can currently make their way through the EU Novel Food regulation process towards markets acceptance, and participate to the so‐called ‘protein shift’ of the next decades. Most of the key microbial biotech players already technically achieved industrial scale and are mostly waiting for regulatory and financial approvals to construct full microbial protein production facilities (e.g. Unibio, Calysta or Solar Foods; Pikaar et al., 2023).

A further challenge for food and feed microbial biotech is to substantiate the environmental benefits that underpin their development rationale. These should not be taken for granted. As for any emerging technology, clever choices need to be made with regard to the combination of feedstock, culture, metabolism and the surrounding deployment context (e.g. access to energy, etc.). A holistic and comparative framework is still required to fairly compare the performances of microbial biotech not only between them, but also against the food or feed production systems these aim to substitute.

To this end, life cycle assessment (LCA) allows to quantify and compare such environmental performances over the many impact categories and unravel the trade‐offs. It provides a good compass in guiding us to the most preferred route, even in an early development stage. For instance, the LCA study of Spiller et al. (2020) demonstrated that a consortium of aerobic heterotrophs only, of microalgae with aerobic heterotrophs and of purple bacteria cultured on potato wastewater to produce microbial proteins, outperformed soybean meal for the human health and ecosystem quality endpoint categories, while soybean meal had a better score for resource depletion. Another study by (Pikaar, Matassa, Bodirsky, et al., 2018) suggested the counterintuitive result that producing microbial proteins with methanotrophs on biomethane derived from residual streams would be less virtuous to global warming and nitrogen pollution than their production of fossil methane.

Yet, recent efforts in harmonizing the LCAs of microbial biotech, applied to the case of waste‐to‐nutrition technologies, nuance their environmental benefits (Javourez et al.,^2024^). Results suggest that the environmental relevance of producing edible ingredients from waste resources through microbial biotech largely relates to the future availability of environmentally friendly energy supplies and on the future direction global food systems will take (in terms of evolution of global animal‐based food demand, deforestation trends, etc.) and that leverages on the process‐design side are of second importance. Notably, the authors conclude that under current circumstances, it is already more virtuous for climate change and nutrient pollution to produce microbial proteins from manure and sludge than to make compost or energy out of it. Yet, for climate change, these conclusions remain valid as long as soybean meal remains embedded with at least 4 kgCO_2‐eq_ per kg_DM_. Such advances in prospective and ex‐ante LCA studies may even call into question the overall relevance of microbial biotech R&D strategies, and help them to identify technological dead‐ends before investments are made. For example, recent work demonstrates that bioprospecting efforts to find compounds in microalgae to mitigate the environmental impacts of the aquaculture sectors are likely worthless, even if undiscovered (Jouannais, 2024).

The same applies to the foreseen socio‐economic implications of microbial biotech deployment. Most of the axenic fermentation options assessed require large‐scale production and sophisticated technologies likely to only promote centre‐of‐the‐plate (or of the feed formulation) changes (Howard, 2022). Smallholder food production and livestock farming represent the livelihood of billions of people worldwide, who will be affected under a scenario of fully automatized large‐scale alternative protein facilities. Moreover, navigating around the ‘cracking‐building’ food chemistry production pattern, food‐ and feed‐oriented axenic fermentation microbial biotech might replicate health burdens associated with (highly) processed ingredients (Gastaldello et al., 2022; Reynolds et al., 2023). On the other hand, symbiotic fermentation‐oriented microbial biotech is more likely to find a position within the landscape of the transition towards sustainable and fair food systems, given the possibility of having small‐scale fermenters for local production (even at the home level).

## WHAT ABOUT GRASSLANDS AS A FEEDSTOCK SOURCE FOR MICROBIAL BIOTECH?

Many microbial biotech solutions are on the table. In the meantime, the EU proposes to repurpose the use of arable lands, and particularly to redefine the role of grassland around the concept of green biorefineries. Grasslands present the best plant‐based protein productivities and regulate nitrification processes (Jørgensen & Lærke, 2016). Separating the juice and the fibre fraction of grass, a variety of value‐added co‐products is obtained (including food and feed items), enabling the possibility to reconsider the role of ruminants as pivotal users of this land cover (grasslands represent 26% of total emerged surfaces; Lü et al., 2022). Green biorefinery schemes cannot only make grass proteins available to monogastrics and convert grasslands into net energy providers (e.g. through anaerobic digestion of protein extraction waste), but also supply microbial biotech with nitrogen and fibres (Pihlajaniemi et al., 2020). A special remark in this context could be the clever usage of biological fixation to compensate for the unbalanced C:N ratio of plant fibres and support microbial biotech shifting away from Haber‐Bosch reactive nitrogen perfusion. We believe that in the coming decades, the ultimate goal of microbial biotechnology should be to develop a fermentative process that utilizes cellulosic materials and dinitrogen to produce valuable outputs, especially proteins for feed and food.

## CONCLUSION: EDUCATION, COMMUNICATION AND PROGRESS BY MEANS OF EXPERIMENTAL TESTBEDS

With as much as 800 kilograms of food ingested per capita per year on average, issues related to food supply, safety, risks and innovations are sensitive and often spark passionate debates. So far, the dominance of conservative discourses and adverse effects of the cautionary approaches of regulatory agencies have limited to tails the promises of microbial biotech towards food and feed production. Yet, to achieve a circular bioeconomy and an eco‐friendly food system, it is vital to be able to make use of the power of microbiomes, including refurnishing soil microbiomes (i.e. soil health) and mimicking their synergistic resource recovery strategies (Shayanthan et al., 2022). Microbiomes can already adequately be managed and provide quality assurance. These facts should reach the public, its lawmakers and regulatory agencies through open science, interactive citizen‐science demonstrations, biotechnology literacy education and communication campaigns. Such actions could lead to a more open vision in society towards microorganisms in general and their beneficial roles in particular. Several decades were needed before it became generally accepted that climate change was due to human activities. The concept that we need to use at large‐scale microbiomes to reach the Sustainable Development Goals is still in its infancy. Experimental testbeds, in which open‐minded innovators, entrepreneurs, regulators and consumers cooperate over extended periods of time to explore the potentials of new feeds and foods based on fermentations, both axenic and natural symbiotic types, are warranted, particularly if we want to progress towards a much more sustainable feed and food supply for the future.

## AUTHOR CONTRIBUTIONS

**Ugo Javourez:** Investigation; validation; writing – original draft. **Silvio Matassa:** Investigation; validation; visualization; writing – review and editing. **Siegfried E. Vlaeminck:** Investigation; validation; writing – review and editing. **Willy Verstraete:** Conceptualization; investigation; validation; writing – original draft.

## FUNDING INFORMATION

This research received no specific grant from any funding agency, commercial or not‐for‐profit section.

## CONFLICT OF INTEREST STATEMENT

None.
